# A reduction of CETP activity, not an increase, is associated with modestly impaired postprandial lipemia and increased HDL-Cholesterol in adult asymptomatic women

**DOI:** 10.1186/1476-511X-10-87

**Published:** 2011-05-24

**Authors:** Eliane S Parra, Aline Urban, Natalia B Panzoldo, Rui T Nakamura, Rogério Oliveira, Eliana C de Faria

**Affiliations:** 1 Department of Clinical Pathology, Lipid Laboratory and Center for Medicine and Experimental Surgery, Faculty of Medical Sciences, University of Campinas, (Rua Tessália Vieira de Camargo), Campinas, (zip code 13084-971), Brazil; 2 Department of Radiology, Faculty of Medical Sciences, University of Campinas, (Rua Tessália Vieira de Camargo), Campinas, (zip code 13084-971), Brazil; 3 Department of Statistics, Mathematic and Statistics Institute, University of São Paulo, (Rua do Matão, 1010), São Paulo, (zip code 05311-970), Brazil

**Keywords:** HDL-cholesterol, cholesteryl ester transfer protein variation, postprandial lipemia and regulation, common carotid IMT

## Abstract

**Background:**

The relationship between CETP and postprandial hyperlipemia is still unclear. We verified the effects of varying activities of plasma CETP on postprandial lipemia and precocious atherosclerosis in asymptomatic adult women.

**Methods:**

Twenty-eight women, selected from a healthy population sample (n = 148) were classified according to three CETP levels, all statistically different: CETP deficiency (CETPd ≤ 4.5%, n = 8), high activity (CETPi ≥ 23.8, n = 6) and controls (CTL, CETP ≥ 4.6% and ≤ 23.7%, n = 14). After a 12 h fast they underwent an oral fat tolerance test (40 g of fat/m^2 ^of body surface area) for 8 hours. TG, TG-rich-lipoproteins (TRL), cholesterol and TRL-TG measurements (AUC, AUIC, AR, RR and late peaks) and comparisons were performed on all time points. Lipases and phospholipids transfer protein (PLTP) were determined. Correlation between carotid atherosclerosis (c-IMT) and postprandial parameters was determined. CETP TaqIB and I405V and ApoE-ε3/ε2/ε4 polymorphisms were examined. To elucidate the regulation of increased lipemia in CETPd a multiple linear regression analysis was performed.

**Results:**

In the CETPi and CTL groups, CETP activity was respectively 9 and 5.3 higher compared to the CETPd group. Concentrations of all HDL fractions and ApoA-I were higher in the CETPd group and clearance was delayed, as demonstrated by modified lipemia parameters (AUC, AUIC, RR, AR and late peaks and meal response patterns). LPL or HL deficiencies were not observed. No genetic determinants of CETP deficiency or of postprandial lipemia were found. Correlations with c-IMT in the CETPd group indicated postprandial pro-atherogenic associations. In CETPd the regression multivariate analysis (model A) showed that CETP was largely and negatively predicted by VLDL-C lipemia (R^2 ^= 92%) and much less by TG, LDL-C, ApoAI, phospholipids and non-HDL-C. CETP (model B) influenced mainly the increment in ApoB-100 containing lipoproteins (R^2 ^= 85% negatively) and phospholipids (R^2 ^= 13%), at the 6^th^h point.

**Conclusion:**

The moderate CETP deficiency phenotype included a paradoxically high HDL-C and its sub fractions (as earlier described), positive associations with c-IMT, a postprandial VLDL-C increment predicting negatively CETP activity and CETP activity regulating inversely the increment in ApoB100-containing lipoproteins. We hypothesize that the enrichment of TG content in triglyceride-rich ApoB-containing lipoproteins and in TG rich remnants increases lipoproteins' competition to active lipolysis sites,reducing their catabolism and resulting on postprandial lipemia with atherogenic consequences.

## Background

The relationship between cholesteryl ester transfer protein (CETP) and lipoprotein metabolism is very complex and, in different metabolic backgrounds, depends largely on the concentration of high-density lipoprotein (HDL) and/or triglyceride-rich lipoproteins (TRL) [1]. It is well established that, during reverse cholesterol transport (RCT) [2], CETP is essential in neutral lipid exchange among lipoproteins [3], and can decrease circulating oxidized low-density lipoprotein (LDL) [4,5]. One of the major mechanisms by which HDL protects against atherosclerosis is the RCT where increased selective uptake of HDL-cholesteryl ester (CE) by scavenger receptor class B type I (SRBI) [6] transfers cholesterol from atherosclerotic lesions macrophages to the liver, decreasing macrophage CE content [5] and excreting cholesterol into the bile, with intravascular lipoprotein remodeling [7].

This mechanism is anti-atherogenic in normolipidemic individuals, but in cases of hypercholesterolemia and mixed hyperlipidemia, CETP can have a pro-atherogenic role, because of the generation of dense, small and atherogenic LDL [8,9]; elevated levels of apolipoprotein B (ApoB)-containing acceptor particles for CETP lead to enhanced transfer of triglycerides (TG) from very-low-density lipoprotein (VLDL) to HDL, with consequent TG enrichment of HDL and abnormal intravascular metabolism, involving reduction in particle size and decrease of HDL-Cholesterol (-C) and ApoA-I levels [10,11].

The importance of plasma CETP in lipoprotein metabolism was demonstrated by the discovery of CETP-deficient subjects with severe hyperalphalipoproteinemia [12]. Genetic CETP deficiency is caused by mutations in the *CETP *gene (OMIM 607322) that is located on chromosome 16q21 [13], and is the most important and common cause of hyperalphalipoproteinemia in the Japanese [14]. It is considered a physiological state of impaired RCT, which may possibly lead to the development of atherosclerosis despite high HDL-C concentrations [15].

Several polymorphisms have been reported in the human CETP locus [16], some of them reducing synthesis of CETP [17-21] and one reducing activity [19]. Family members have in their plasma high levels of HDL-C [17-19] known to be a negative risk factor for coronary artery disease [22-24].

The most commonly studied polymorphism is in the TaqI site (TaqIB), which is a silent base change of guanine to adenine nucleotide substitution at the 279^th ^nucleotide position in the first intron of the CETP gene [16]. In the general population, the TaqIB polymorphism is associated with variations of both CETP mass and activity and HDL-C concentrations, and the less common *B2B2 *genotype (absence of the TaqIB restriction site) has been associated with increased HDL-C levels and decreased CETP activity and levels [25-27].

The I405V polymorphism is a transition of adenine to guanine in position +20206 of exon 14 which leads to a missense mutation with the substitution of valine for isoleucine in position 405 of the protein [28]. In the homozygous form for the rarest allele (V/V genotype) the I405V polymorphism is associated with a reduction in CETP activity, and with increased levels of HDL-C [27,28].

CETP deficiency and inhibition studies, in animals and humans, have provided conflicting results. Pharmacologic CETP inhibition has increased HDL-C and reduced atherosclerosis in rabbit models [29]. In humans, CETP deficiency has been associated with both increased and decreased coronary heart disease (CHD) risk [29,30]. The CETP inhibitors JTT-705 and torcetrapib have been shown to effectively reduce CETP activity in humans and raise HDL-C, although the effect of this class of compounds on atherosclerosis and CHD risk remains unclear [31,32]

CETP deficiency is overlooked on its actions on postprandial lipemia, a state that has been associated with quantitative and qualitative alterations of the lipid profile, positively related to the progression of cardiovascular risk [33]. The lipoproteins involved predominantly small chylomicrons and VLDL remnants that may go through the vessel wall [34].

TRL are converted into remnant lipoproteins after a gradual hydrolysis process through the action of the lipoprotein lipase (LPL) [35]. The postprandial lipemia elicits diverse metabolic, oxidative and atherogenic events including chylomicron remnant production, formation of small LDL particles and reduction of the concentration of cardioprotective fractions of HDL. The postprandial pro-atherogenic effects on the metabolism of TRL can be direct on the vessel wall, due to accumulation of these particles [9], or indirect, through changing status of inflammatory aspects such as the activation of leukocytes and of endothelin, as well as the activation of the complement system [36].

During lipolysis of postprandial TRL, an excess of surface components (Apo, unesterified cholesterol and phospholipids (PL)) is generated and sequesters to HDL potentially via the action of hepatic lipase (HL) and PL transfer protein (PLTP), thereby increasing the total circulating HDL pool and enhancing the transformation of small HDL_3 _to large CE-rich HDL_2 _particles. Equally, such transfer is accelerated under postprandial conditions with CE enrichment of TRL particles, and transient transformation of CE-enriched HDL into TG-enriched particles which become a substrate for HL. This results in modulation of the size of the HDL pool [37].The effects of CETP on postprandial lipemia in humans [8,33,37] and in experimental animals [38,39] have been broadly explored, but controversies over the results persist, and can be seen in the two studies by Ritsch, 1997 [40] and Ai, 2009 [41], where CETP deficiency coexisted with impaired lipemic status.

Other alterations that may occur during this period can be related mainly to the activities of proteins and enzymes like CETP, HL and LPL [42].

The ApoE is a polymorphic protein, and one of the major protein constituents of TRL. It serves as a high affinity ligand for several hepatic lipoprotein receptors, including the LDL receptor and the LDL receptor-related protein. By interacting with these receptors, ApoE mediates the clearance of TRL and their remnants from the circulation [43].

The ApoE gene is located on chromosome 19q13.2, consisting of four exons and three introns, and the common variations at the ApoE gene locus that create the ApoE-ε2, ε3 and ε4 isoforms are major determinants of plasma lipid and lipoprotein levels [16]. Compared with the most common ε3 isoform, carriers of the ε2 isoform have lower levels of LDL-C, total cholesterol and ApoB and higher levels of plasma TG [44,45], whereas ApoE-ε4 is associated with higher plasma total cholesterol, LDL-C and ApoB [16,44].

High-resolution ultrasonography is a non invasive technique that allows changes in the arterial wall of carotid and femoral arteries to be seen and measures the thickness of the arterial intima-media complex [46]. Cross-sectional and population studies indicating an association between carotid intima-media thickness (c-IMT) cardiovascular disease, predominantly coronary artery disease [47], and risk [48] are widely described in the literature. More importantly, in prospective studies c-IMT was able to predict coronary artery disease [49].

The objective of this study was to determine the relationships between deficient, non altered and increased CETP activity of asymptomatic adult women and postprandial lipemia. These variations could provide the opportunity to elucidate the metabolic role of CETP on the postprandial state in humans, considering that in experimental animals this effect has been largely studied [38,39]. Also, the mean common c-IMT, as a marker of precocious atherosclerosis, was tested for a relationship with metabolic markers of the postprandial state.

## Methods

### Experimental protocol

Twenty-eight volunteer women, asymptomatic, normolipidemic as defined by National Cholesterol Education Program [50], aged from 20 to 63y were selected and classified in 3 groups according to CETP activity values obtained in a previously studied normolipidemic population sample (n = 148, unpublished data). The CETP deficiency (CETPd) group (n = 8), characterized by CETP activity at the 10^th ^percentile values or below, CETP activity ≤ 4.5%; the control (CTL) group (n = 14), with CETP activity above the respective 10^th ^and below their 90^th ^percentile values, CETP activity ≥ 4.6 and ≤ 23.7%; and the subjects with high levels of CETP activity (CETPi group, n = 6), selected through activity above their respective 90^th ^percentile, CETP activity ≥ 23.8% answered a detailed questionnaire aimed at determining: cardiovascular diseases (presence of angina pectoris, myocardial infarction, coronary insufficiency, the history of coronary revascularization procedures, coronary angioplasty and coronary grafting bypass), diabetes mellitus, cigarette smoking, family history of coronary heart disease, hypertension, alcohol consumption, sedentariness, menopause accompanied with use of hormone replacement therapy, use of contraceptive pills and other related drugs, body mass index (BMI)>30 kg/m^2^, liver and kidney disease. The volunteers were excluded if they fulfilled one of the characteristics above and showed the presence of ApoE-ε2 isoform, because it is associated with high fasting levels of plasma triglycerides.

All selected individuals underwent an oral fat tolerance test. The test began by venous puncture after a 12-h fast followed by ingestion of a milkshake prepared with lactose-free powdered milk (NAN^®^, Nestlé, São Paulo, Brazil). The liquid meal contained fat (25%), dextromaltose (55%), protein (14%), and vitamins and minerals (6%), providing 40 g of fat per square meter of body surface, and was given over a 10 min period. Serial blood samples were collected at 2, 4, 6, and 8 h after the ingestion.

### Determination of carotid intima-media thickness

The common c-IMT was measured by ultrasonography using the HDI 500 Ultrasound System equipment (ATL Ultrasound, Bothell, WA, USA), with a 7- to 12-MHz color Doppler probe. c-IMT was calculated as the mean of five measurements in the far wall from the left and right common carotid arteries according to a standardized method [51,52]. Individual results were expressed in millimeters as an average of the left and right c-IMT.

### Plasma Lipids, Lipoproteins, and Apolipoproteins

Measurements were performed on samples from all time points. TRL, at a density lower than 1.006 g/L, were isolated by sequential ultracentrifugation for 16 h at 4°C and 40.000 rpm in a Beckman centrifuge (model L5-75B, Beckman, Palo Alto, CA, USA). Cholesterol and TG in serum and in TRL particles were measured by enzymatic-colorimetric methods (Hitachi Modular, Roche, Mannheim, Germany); LDL-C and HDL-C were analyzed by homogeneous direct methods (Roche Diagnostic Mannheim, Germany). Fasting HDL sub fractions (HDL_2 _and HDL_3_) were obtained by sequential micro-ultracentrifugation of the supernatants after precipitation of lipoprotein containing ApoB-100 with 5% dextran sulfate with posterior quantification of cholesterol and TG by enzymatic-colorimetric methods. ApoA-I and ApoB-100 were analyzed by nephelometry. Non-esterified fatty acids (NEFA), PL, and free cholesterol (FC) were determined through enzymatic-colorimetric methods (Wako, Osaka, Japan). CE were calculated by multiplying the difference of total cholesterol and FC by 1.67 as recommended [53].

### Lipases and Lipid Transfer Proteins

LPL and HL activities were measured in fasted post-heparin plasma samples, collected 15 min after the intravenous administration of heparin, at 100 U/kg body weight, on the basis of fatty acid release, by using a radiolabeled triolein emulsion as the substrate and NaCl (1 M) as the LPL inhibitor; the results were expressed as nanomoles of NEFA per milliliter per hour [54].

CETP activity was determined by an exogenous assay that measures the transfer of radiolabeled CE between a "normal" donor pool of ^14^CE-HDL and an unlabeled acceptor mixture of VLDL plus LDL over 4 h by using plasma as the CETP source, and results are expressed as percentage of CE transferred [55]. The PLTP was measured by an exogenous radiometric method using PL liposomes as the substrate [56].

Assays for CETP, PLTP and lipase activities were conducted in triplicate. Interassay coefficients of variation were 12%, 15%, 9%, and 8% respectively for CETP, PLTP, LPL, and HL.

### CETP and Apolipoprotein E genotyping

Some gene polymorphisms of interest, such as ApoE and CETP were determined using RT-PCR.

ApoE genotype was performed according to Emi, 1988 [57] and CETP I405V and TaqIB CETP polymorphisms were detected as described by Gudnason, 1999 [58] and Fumeron, 1995 [59].

### Statistical Analysis

All statistical evaluations were performed by a trained statistician from the institution and using the SAS software.

TG, FC, NEFA, CETP, TRL-C and TRL-TG were log transformed variables.

Wilcoxon, Mann-Whitney and Chi-Square tests were performed for comparisons between groups or within each group and, when necessary, ANCOVA was used to adjust the variables.

Spearman's correlation with Bonferroni's correction related the variables.

Observational analysis was used for the postprandial curves. The trapezoidal method estimated the area under the curve (AUC) and the area under the incremental curve (AUIC). Slopes of the individual curves were determined by linear regression analysis and expressed as the acquisition rate (AR), from 0 h to TG peak, and as the removal rate (RR), from peak to the time with the lowest TG concentration as the summary measurements of curves. Late peaks were defined as significant increased point values when compared to the previous time point and their frequencies were calculated.

A multiple linear regression analysis was performed to establish the predictors of CETP (model A) using as independent variables age, waist circumference (WC), BMI, TG, VLDL-C, TRL-TG, TRL-C, ApoA-I, ApoB100, NEFA, PL, LPL, PLTP, CETP, HL, and AUCs, AUICs, AR and RR.

Secondly, a hierarchical multiple linear regression analysis with stepwise criteria for selection of variables was used to assess the influence of CETP on postprandial lipemia (model B); the dependent variables were: TG, TRL-TG, TRL-C with the corresponding AUC, AUIC, AR and RR. Results are expressed as partial coefficients of determination (*R*^2^) that represent percentages of variation in the dependent variables.

The significance level used was ≤5% and borderline levels were >5% p value p ≤ 10%.

### Ethical aspects

All subjects gave written informed consent. The research protocol was approved by the research ethics committee of the School of Medicine of the State University of Campinas, São Paulo.

## Results

### Subjects'characteristics

#### Fasting parameters

Clinical data are shown in Table 1. The CETPd women were older and because of this all the comparisons or correlations in this study were statistically corrected for age. c-IMT was higher in CETPd women, but all the other anthropometric variables were similar in all groups. The subjects in this study are asymptomatic adult women presenting the recommended ranges of blood pressure, BMI, and WC. No differences were found in CETPi women as compared to controls.

**Table 1:** Clinical and genetic characteristics and common carotid IMT of women in the studied groups

Variables/Groups	CETPd (n = 8)	CETPi (n = 6)	CTL (n = 14)
Age (y)	**52.5 ± 9.5^a^**	**33.8 ± 6.5**^b^	**28.6 ± 8.4^c^**
BMI (Kg/m^2^)	25.4 ± 4.4	23.0 ± 2.2	21.8 ± 1.9
WC (cm)	78.2 ± 14.5	74.7 ± 4.5	72.6 ± 6.9
SBP (mmHg)	116.2 ± 11.9	115.8 ± 4.9	110.8 ± 8.1
DBP (mmHg)	72.5 ± 8.9	76.7 ± 5.2	73.0 ± 7.3
Mean c-IMT (mm)	0.7 ± 0.1	0.6 ± 0.1	0.5 ± 0.1
CETP TaqIB - B1 allele %	50.0 (7)	62.5 (5)	50.0 (12)*
CETP TaqIB - B2 allele %	50.0 (7)	37.5 (3)	50.0 (12)*
CETP I405V - I allele %	64.3 (9)	37.5 (3)	41.7 (10)*
CETP I405V - V allele %	35.7 (5)	62.5 (5)	58.3 (14)*
ApoE-ε(3/3+3/4) %	100.0	100.0	93.0 *

Data as mean ± SD; defined by levels of CETP activity (diminished (d), increased (i) and controls (CTL); BMI, body mass index; WC, waist circumference, SBP, systolic blood pressure; DBP, diastolic blood pressure; c-IMT, mean common carotid intima-media thickness. Mann-Whitney and X^2^-square tests *(NS); a vs b, p ≤ 0.0001; a vs c p < 0.004; p values corrected for age by ANCOVA Significant differences are shown in bold CETP TaqIB and I405V polymorphisms: frequency of B1 or B2 alleles and of V or I alleles.

Regarding the biochemical characteristics (Table 2), CETP activity of CETPi and CTL were 9 and 5.3 higher as compared to the CETPd. Concentrations of cholesterol and TG in HDL and sub-fractions were higher in the CETPd group and statistically different from CTL, which is expected and secondary to CETP deficiency. As compared to the CETPd group, HDL-C was 1.6 and 1.5 higher, HDL_2_-C, 1.6 and 1.4 higher, HDL_3_-C, 1.6 and 1.5 higher and ApoAI, 1.4 and 1.3 higher in CETPi and CTL respectively. TG enrichment of HDL subfractions was observed: HDL_2_-TG, 1.3 and in HDL_3_-TG, 1.3 higher as compared to CETPi (HDL_2_-TG) and to CTL (HDL_3_-TG). HDL from the CETPd group may be dysfunctional due to the increased triglycerides content. NEFA were higher in CETPi as compared to CETPd, maybe due to a faster TRL catabolism in the first, but NEFA increment was higher in CETPd as compared to CETPi (table 3). No differences were found in CETPi women as compared to controls, indicating their similarities.

**Table 2:** Fasting biochemical parameters of women in the studied groups

Variables/groups	CETPd (n = 8)	CETPi (n = 6)	CTL (n = 14)
CETP (%)	3.1 ± 1.3*	26.8 ± 1.5*	15.3 ± 5.1*
HL (nmol NEFA/mL/h)	1584.1 ± 723.1	2066.8 ± 533.5	2090.0 ± 1033.1
LPL (nmol NEFA/mL/h)	3189.2 ± 2364.1	2494.8 ± 372.6	2387.2 ± 989.1
PLTP (%)	8.3 ± 5.2	8.6 ± 2.9	8.8 ± 4.2
FC (mg/dL)	54.0 ± 2.6	40.4 ± 12.0	49.2 ± 16.1
CE (mg/dL)	146.0 ± 16.4	110.3 ± 26.0	105.9 ± 24.2
C (mg/dL)	195.1 ± 18.3	150.7 ± 34.2	158.8 ± 31.5
TG (mg/dL)	78.0 ± 24.6	55.8 ± 15.1	65.6 ± 29.9
HDL-C (mg/dL)	**79.1 ± 16.0^a^**	**49.7 ± 7.0^b^**	**52.1 ± 8.7^c^**
HDL_2_-C (mg/dL)	**17.1 ± 5.1^d^**	10.8 ± 3.1	**13.3 ± 2.0^e^**
HDL_2_-TG (mg/dL)	**7.9 ± 3.6^f^**	**5.7 ± 4.3^g^**	6.3 ± 5.2
HDL_3_-C (mg/dL)	**57.1 ± 9.0^h^**	**35.8 ± 5.5^i^**	**39.1 ± 7.1^j^**
HDL_3_-TG (mg/dL)	**20.8 ± 6.2^k^**	14.6 ± 4.1	**15.6 ± 12.6^l^**
LDL-C (mg/dL)	110.2 ± 19.8	93.0 ± 22.6	95.1 ± 23.2
LDL-C/ApoB-100	**1.3 ± 0.1^m^**	**1.4 ± 0.1^n^**	1.3 ± 0.1
VLDL-C (mg/dL)	15.5 ± 4.9	11.0 ± 3.0	13.2 ± 6.0
NHDL-C (mg/dL)	118.0 ± 18.7	101.0 ± 28.1	106.7 ± 26.7
NEFA (mmol/L)	**0.4 ± 0.2°**	**0.7 ± 0.2^p^**	0.6 ± 0.4
PL (mg/dL)	**270.3 ± 56.8^q^**	**197.0 ± 31.9^r^**	212.1 ± 41.1
ApoA-I (mg/dL)	**191.9 ± 20.6^s^**	**134.3 ± 16.7^t^**	**147.6 ± 28.8^u^**
ApoB-100 (mg/dL)	84.4 ± 12.3	66.5 ± 13.8	72.0 ± 18.9

Data as mean ± SD; *groups defined by CETP activity: CETPd vs CTL, (p ≤ 0.00003), CETPi vs CTL, (p ≤ 0.00001) and CETPd vs CETPi, (p ≤ 0.00001); CETP, cholesteryl ester transfer protein activity; HL, hepatic lipase activity; LPL, lipoprotein lipase activity; PLTP, phospholipid transfer protein activity; FC, free cholesterol; CE, cholesteryl ester; C, total cholesterol; TG, triglycerides; HDL_2 _and HDL_3_, high-density lipoprotein sub fractions; LDL-C, low-density lipoproteins cholesterol; VLDL-C, very-low-density lipoprotein cholesterol, NHDL-C non-high-density lipoprotein cholesterol; NEFA, non-esterified fatty acids; PL, phospholipids; Apo, apolipoprotein; p values by Mann-Whitney with ANCOVA corrections for age: CETPd vs CTL; a vs c, p ≤ 0.010; d vs e, p ≤ 0.010; h vs j, p ≤ 0.01; k vs l, p ≤ 0.002; s vs u, p ≤ 0.030; CETPd vs CETPi - a vs b, p ≤ 0.010; f vs g, p ≤ 0.010; h vs i, p ≤ 0.010; m vs n, p ≤ 0.030; o vs p, p ≤ 0.030; q vs r, p ≤ 0.030; s vs t, p ≤ 0.010. Significant and borderline differences are shown in bold.

**Table 3:** Significant differences of AUC, AUIC and RR in the studied groups

Variables/groups	CETPd (n = 8)	CETPi(n = 6)	CTL (n = 14)
RR TG	**-**5.1 ± 24.0	**59.6 ± 33.3**^a^	**15.1 ± 13.0**^b^
AUC TRL-TG	**1632.0 ± 711.2^c^**	**373.2 ± 112.3^d^**	1102.2 ± 462.1
AUIC TRL-TG	**454.3 ± 262.1^e^**	**196.6 ± 114.3^f^**	217.9 ± 115.2
RR TRL-TG	**-14.4 ± 17.8^g^**	**11.2 ± 6.6^h^**	**-19.2 ± 25.3^i^**
AUC TRL-C	**143.2 ± 67.9 ^j^**	**53.7 ± 17.7^l^**	**63.2 ± 29.0^m^**
AUIC NEFA	**-0.2 ± 1.7^n^**	**-1.6 ± 0.2^n^**	**-2.5 ± 1.6°**

Data as mean ± SD. AUC (mg/dL h), areas under curves; AUIC(mg/dL h), area under the incremental curve; AR (mg/dL), acquisition rate, RR (mg/dL), removal rate; AR (mg/dL), acquisition rate: not shown, NS; TG, triglycerides; TRL-C or TRL-TG, cholesterol or triglyceride in triglyceride-rich lipoproteins; NEFA, non-esterified fatty acids. Mann-Whitney and ANCOVA for corrections for age; CETPi vs CTL: a vs b, p ≤ 0.001; CETPd vs CETPi: c vs d, p ≤ 0.040; e vs f, p ≤ 0.048; g vs h, p ≤ 0.004; j vs l, p ≤ 0.021 and CETPd vs CTL: g vs i, p ≤ 0.045; j vs m, p ≤ 0.040; n vs o, p ≤ 0.043. Significant differences are shown in bold

As compared to CTL or CETPi, LPL and HL were 30% higher and lower respectively in the CETPd, but it did not reach significancy. PLTP was similar among the groups.

#### Genotyping and CETP activities

We excluded the effect of the allele 2 of ε3/ε2/ε4 polymorphisms of ApoE. The prevalent ApoE-ε(3/3+3/4) genotype, equally distributed among the 3 groups, also was not the cause of fat intolerance. Table 1 shows the similar frequency of TaqIB and I405V CETP allelic distribution among the groups, indicating that these CETP mutations were not the cause of CETP deficiency.

The CETP activity (Table 4) of V and B2 alleles from CETP I405V and TaqIB polymorphisms respectively indicate the lowest activities in the CETPd that are independent of the polymorphism. CETPi had the highest values and CTL intermediate ones.

**Table 4:** Plasma CETP activities of CETP TaqIB and I405V polymorphisms in the studied groups

Genotypes		CETPd (n = 8) (% transfer)	CETPi (n = 6) (% transfer)	CTL (n = 14) (% transfer)
CETP TaqIB	B1B1	2.4 ± 1.1 (3)	26.6 ± 0.9 (2)	15.4 ± 0.0 (1)
	(B1B2+B2B2)	**3.3 ± 1.3 (4)^a^**	**26.4 ± 3.2 (2)^b^**	**16.0 ± 5.2 (11)^c^**

CETP I405V	II	3.5 ± 0.7 (3)	-	11.8 ± 5.1 (2)
	(IV+VV)	**2.4 ± 1.5 (4)^d^**	**26.5 ± 1.8 (4)^e^**	**16.7 ± 4.7(10)^f^**

Data as mean ± SD (n). **- **= no II in CETPi. CETP activity; p values by Mann-Whitney with ANCOVA corrections for age: Absence of B2 allele: a vs b p ≤ 0.087; Presence of B2 allele: a vs b p ≤ 0.050, b vs c p ≤ 0.027, a vs c p ≤ 0.005. Presence of V allele: d vs e p ≤ 0.025; d vs f p ≤ 0.002; e vs f p ≤ 0.008. Significant differences are shown in bold.

#### Postprandial lipemia

**Figure 1:**
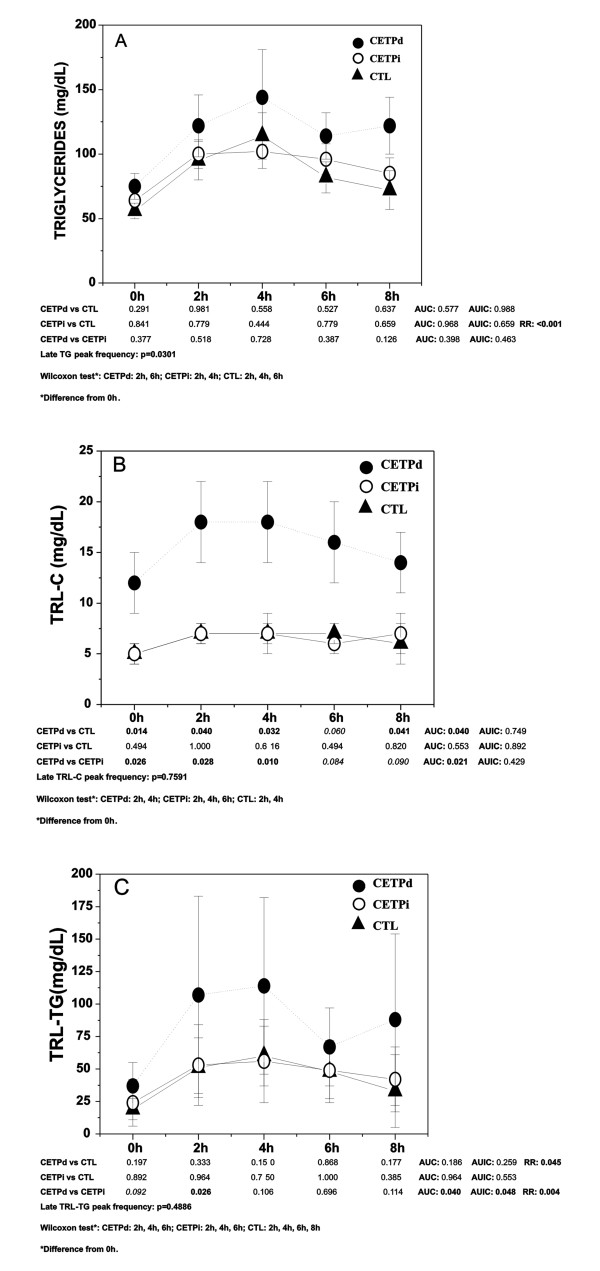
**Postprandial lipemia of women with diminished or increased CETP activity and in controls**. Data as mean ± SEM; correction for age difference was done using the ANCOVA test; the groups were defined by levels of CETP activity: diminished (d), increased (i) and controls (CTL); CETPd, solid circles, CETPi, open circles and triangles, CTL. p values represent: point-to-point differences, AUC, AUIC, AR and RR by Mann-Whitney; p values adjusted for age; postprandial responses in each group by Wilcoxon test and frequency of late peaks by Chi-Square test.

As shown in Figure (1A, B and 1C) and in Table 3 moderate deficiency of CETP delayed plasma TRL clearance.

Figure 1-A shows that the curve displayed a higher frequency of late TG peaks in CETPd (p ≤ 0.030) as compared to CTL (late TG peak at 8 h after the meal intake). TG removal rate was increased in CETPi as compared to CTL.

An increased AUC-TRL-C (Figure 1B) was observed in CETPd as compared to CETPi and to CTL. Significant differences were observed point-to-point for TRL-C between CETPd and CTL (except at 6 h, borderline difference).

In Figure 1C, TRL-TG was marginally increased at 0 h and at 2 h in CETPd as compared to CETPi. TRL-TG AUC and AUIC were increased in CETPd as compared to the CETPi; TG removal rate was reduced in CETPd as compared to the other two groups.

Fat meal intolerance is furthermore characterized in Figure 1 and in Table 3 where the significantly different lipemia parameter values are shown.

In CETPd, AUC-TRL-C was 2.3 and 2.6 times higher than in CTL and CETPi respectively. RR-TG in CETPd was reduced by 3 to 12 times less as compared to CTL and CETPi respectively and RR-TRL-TG was 1.3 lower than in CETPi. AUIC-NEFA was 12 times higher in CETPd as compared to CTL and 8 times higher in CETPd vs CETPi.

Observing in Figure 1 the Wilcoxon' test responses to the high fat meal and by comparing the baseline values to each point along the period we did not identify higher lipemia responses in the CETPd group, and all groups were apparently similar.

#### Carotid atherosclerosis and correlations

The cardiovascular repercussion of the phenotypes was measured by c-IMT. Carotid measurements after correction for age were similar among the groups, indicating the well known age effect on c-IMT. The CETPd women showed stronger correlations of c-IMT with the lipemia parameters: TG and TRL at 0, 4, and 6 h. There was a positive correlation with NEFA at 2 h in CETPi, suggesting the presence of an association of the variables during postprandial lipemia. The CTL group presented one weaker, positive, and more physiological association of AR-TRL-TG with c-IMT (Table 5)

**Table 5:** Significant correlation coefficients between common carotid IMT and biochemical parameters in the studied groups

Groups	c-IMT versus	p values	r
CETPd (n = 8)	**TRL-TG 4 h**	**0.050**	0.750
	**TRL-C 0 h**	**0.052**	**0.707**
	**TRL-C 6 h**	**0.006**	**0.862**
			
CETPi (n = 6)	**NEFA 2 h**	**0.038**	**0.836**
			
CTL (n = 14)	**AR-TRL-TG**	**0.036**	**0.608**

Spearman's correlation with Bonferroni's correction: r and p values are corrected for age; c-IMT, carotid intima-media thickness; TG, triglycerides; TRL-C or TRL-TG, cholesterol or TG in triglyceride-rich lipoproteins; NEFA, non-esterified fatty acids; AR-TRL-TG, acquisition rate of TRL-TG.

#### Multivariate analysis

In view of the pattern of fat intolerance observed in the CETPd group, we performed the analysis of the influence of CETP on metabolic and clinical variables and next the influence of lipemia markers on CETP in this group (Table 6).

**Table 6:** Influences of postprandial lipemia on CETP activity and of CETP activity on postprandial variables in CETPd women

Independent variables	Dependent variables	p values	Partial R²
Model A (n = 8)			
	CETP		
-ApoA-I 4 h		0.0081	0.0051
-AUC-VLDL-C		<0.0001	0.9217
LDL-C 8 h		0.0037	0.0566
NHDL-C 0 h		0.0189	0.0011
PL 2 h		0.0073	0.0003
-TG 4 h		0.0094	0.0153

Model B (n = 8)			
CETP			
	PL 6 h	0.0020	0.1284
	-AIUC-ApoB-100	0.0158	0.8463

**A **Linear regression fitting controlled for age and using the stepwise method; dependent variable, CETP in CETPd

group and the independent variables were age, WC, BMI, TG, VLDL-C, TRL-TG, TRL-C, ApoA-I, ApoB100, NEFA, PL and corresponding AUC, AUIC, AR and RR and LPL, PLTP, CETP, HL.

**B **Linear regression fitting controlled for age and using the stepwise method; independent variable, CETP in CETPd

group; dependent variables were TG, TRL-TG, TRL-C, AUC, AUIC, AR and RR. CETP, cholesteryl ester transfer protein; Apo, apolipoprotein; AUC, area under curve; AUIC, area under the incremental curve; VLDL-C, very-low-density lipoprotein cholesterol; LDL-C, low-density lipoprotein cholesterol; NHDL-C non-high-density lipoprotein cholesterol; PL, phospholipids, TG, triglycerides.

We observed that CETP is mainly and negatively regulated by VLDL-C (92%), by TG (1.50%), and by ApoA-I (0.50%); the regulation by LDL-C was positive (5.66%).

Investigating CETP as an independent variable, ApoB-100 containing lipoproteins were explained inversely by 85% and PL 6 h positively by 13%.

## Discussion

We determined the relationship between CETP activity and postprandial lipoprotein metabolism, measuring TRL after a high fat meal in healthy women with moderate reduction of activities of CETP, with increased CETP activities, and in controls.

The morphology of the TG curve displayed a higher frequency of late peaks in CETPd as compared to CTL. In CETPd after the oral fat-load, as compared to CTL, cholesterol and TG in TRL measured as AUC and AUIC were remarkably increased showing that these individuals had slower clearance of TRL as compared to the other groups. These differences observed in TRL patterns suggest that CETP deficiency worsens lipemia and higher activity restores the clearance capacity to control levels.

The cardiovascular repercussion of the phenotypes were observed by positive correlations of c-IMT with TRL-C (0 h), TRL-TG (4 h) and TRL-C (6 h) in CETPd group, indicating delayed clearance of TRL in this group and a higher pro-atherogenic response.

In order to investigate TG intolerance caused by a variety of factors including heterozygous LPL-deficiency [60], insulin resistance, noninsulin-dependent diabetes mellitus (NIDDM) [61], and ApoE isoforms [62] we determined ApoE genotypes, and HL and LPL activities.

These factors did not explain the CETPd phenotype because the results showed similar distributions of the allele ε2 of ApoE and ApoE-ε(3/3+3/4) genotype and similar lipases activities (Tables 1 and 2).

Ritsch (1997) [40] and Ai (2009) [41] reported 2 cases of CETP deficiency, a woman (65y) and a man (40y) with increased postprandial lipemia, but in these cases, the genotype ApoE-ε3/2 and 4/2 phenotypes, respectively, could explain, at least in part, the cause of TG intolerance. It is noteworthy that these studies are case reports of individuals with genetic CETP deficiency and with ApoE-ε3/2 and 4/2 phenotypes, respectively, and in Ai et al [41] the patient was hyperlipidemic.

In the present study, however, the ApoE phenotypes are similar among all groups, and our volunteers are adults (37y ± 13y), normolipidemic, non hypertensive, with no genetic CETP primary deficiencies and with anthropometric and biochemical parameters within the reference ranges. The impaired postprandial lipemia state of CETPd women (TG peak = 150 (ours) or 410 [40] or 174 [41] mg/dL), as compared to CETPi or CTL is not as alarming as in those studies. High HDL-C concentrations characterized a paradoxal phenotype, along with fat intolerance and this was seen in the other two studies as well: (HDL-C: 80 (ours) or 172 [40] or 184 [41] mg/dL). Positive history of diabetes and the presence of insulin resistance were excluding criteria in our group.

Although these papers do not give us support to explain the mechanisms involved in our study, they are, up to now, the only studies that showed similar results to ours. The increased postprandial lipemia found in the CETPd, despite being moderate, goes against most studies [8,33,37-39] that show improved postprandial lipemia related to CETP deficiency, due to either genetic [9,38,39,63,64] or drug [37] action.

The multivariate analysis (Table 6) to determine CETP regulation indicated that CETP predicted mainly inverse increments of ApoB-100 postprandial lipemia, acting as an anti-atherogenic protein (Model B). To our knowledge, no other studies described CETP modulation in human CETPd individuals. CETP was also determined largely by increments of VLDL-C (negative) and by LDL-C (positively) as described earlier (Model A).

High HDL-C levels usually signal a metabolic situation with functioning TG clearing capacity. However, in this study, high HDL-C coexisted with increased postprandial lipemia. CETP deficiency results in a low LDL and high HDL phenotype including ApoE-rich, and large HDL that could provide ApoE to chylomicron/VLDL particles during lipolysis in the postprandial state, accelerating remnant lipoprotein uptake by the liver. However, low CETP activity leading to ApoB-containing lipoproteins TG enrichment increases lipoprotein competition to lipolysis sites with consequent lipemia augmentation, even though a reduction in receptor mediated remnant uptake could also be present.

In opposition to this study, a delayed TRL clearance was seen in several studies using mice [38,39] and humans [8,33,37], where CETP activity delayed TRL clearance, but CETP deficiency improved this state.

Since no secondary causes for CETP deficiency [17-21] were found, genetic CETP TaqIB and I405V polymorphisms were examined, but the allelic frequencies as compared to controls were similar (Table 1).

There is debate on whether CETP inhibition will reduce cardiovascular disease risk [65]. In this study the cardiovascular repercussion of the two phenotypes was measured by c-IMT. Although no differences of c-IMT were detected, different correlations were observed: in CETPd the association was positive with TRL. A subfunctional CETP leads to an increased HDL pool. It is described that these particles are dysfunctional in cholesteryl ester exchanging capacity with TG. Then CETP deficiency could contribute to TG intolerance, even without a disadvantage such as an ApoE-ε2 allele.

The moderate CETPi group, as shown in this study, did not modify postprandial lipemia, probably due to the CETP dependence on the TRL pool size shown.

One point that should be corrected in future studies is the small number of participants in this study. This limitation was caused by the many difficulties encountered in selecting healthy individuals with low and high CETP phenotypes for postprandial measurements. Nevertheless, our results help to understand the postprandial state. It highlighted new aspects of the effects of CETP *in vivo *on human TRL metabolism that were up to now rarely studied.

Further postprandial experiments in a larger group of individuals with CETP variations is a goal in our laboratory and will help to shed light on a better understanding of CETP action and its atherosclerotic postprandial consequences.

## Conclusion

In this study a reasonable HDL-C increase caused by moderate CETP deficiency coexisted with increased postprandial lipemia. A subfunctional CETP can lead to an increased HDL pool, but these particles are dysfunctional, with less CE being exchanged for TG. We hypothesize that the enrichment of TG content in ApoB-containing lipoproteins and in remnants increases lipoprotein competition to active lipolysis sites, reducing their catabolism and bringing on postprandial lipemia.

## List of abbreviations

Apo: apolipoprotein; AR: acquisition rate; AUC: area under the curve; AUIC: area under the incremental curve; BMI: body mass index; CE: cholesteryl ester; CETP: cholesteryl ester transfer protein; CETPd: CETP deficiency group; CETPi: high levels of CETP activity group; c-IMT: carotid intima-media thickness; CHD: coronary heart disease; CTL: control group; HDL: high-density lipoprotein; HDL-C: HDL-Cholesterol; HL: hepatic lipase; LDL: low-density lipoprotein; LPL: lipoprotein lipase; NIDDM: noninsulin-dependent diabetes mellitus; NEFA: non-esterified fatty acids; PL: phospholipids; PLTP: phospholipid transfer protein; RCT: reverse cholesterol transport; RR: removal rate; SR-BI: scavenger receptor class B type I; TG: triglycerides; TRL: triglyceride-rich lipoproteins; VLDL: very-low-density lipoprotein; WC: waist circumference

## Competing interests

The authors declare that they have no competing interests.

## Authors' contributions

ESP worked hard in the data analysis and manuscript preparation along with editing it. AU discussed the design of the study, participated in the collection of data and in the first draft of the manuscript; NBP reviewed critically and corrected the manuscript and its English several times. RN performed carotid ultrasonography; RO was the responsible for the statistical analyses. ECF coordinated this work at all times, created the design of the study, and implemented it in the clinical yard. She also analyzed the methods, results, and statistical data and prepared the final manuscript. All authors read and approved the manuscript.
